# Late treatment-related mortality versus competing causes of death after allogeneic transplantation for myelodysplastic syndromes and secondary acute myeloid leukemia

**DOI:** 10.1038/s41375-018-0302-y

**Published:** 2018-12-20

**Authors:** Johannes Schetelig, Liesbeth C. de Wreede, Michel van Gelder, Linda Koster, Jürgen Finke, Dietger Niederwieser, Dietrich Beelen, G. J. Mufti, Uwe Platzbecker, Arnold Ganser, Silke Heidenreich, Johan Maertens, Gerard Socié, Arne Brecht, Matthias Stelljes, Guido Kobbe, Liisa Volin, Arnon Nagler, Antonin Vitek, Thomas Luft, Per Ljungman, Ibrahim Yakoub-Agha, Marie Robin, Nicolaus Kröger

**Affiliations:** 1 Universitaetsklinikum Dresden, Dresden, Germany; 2 DKMS Clinical Trials Unit, Dresden, Germany; 3 Leiden University Medical Center, Leiden, The Netherlands; 4 University Hospital Maastricht, Maastricht, The Netherlands; 5 EBMT Data Office Leiden, Leiden, The Netherlands; 6 University of Freiburg, Freiburg, Germany; 7 University Hospital Leipzig, Leipzig, Germany; 8 University Hospital, Essen, Germany; 9 GKT School of Medicine, London, UK; 10 Hannover Medical School, Hannover, Germany; 11 University Hospital Eppendorf, Hamburg, Germany; 12 University Hospital Gasthuisberg, Leuven, Belgium; 13 Hopital St. Louis, Paris, France; 14 Deutsche Klinik für Diagnostik, Wiesbaden, Germany; 15 University of Münster, Münster, Germany; 16 Heinrich Heine Universität, Düsseldorf, Germany; 17 HUCH Comprehensive Cancer Center, Helsinki, Finland; 18 Chaim Sheba Medical Center, Tel-Hashomer, Israel; 19 Institute of Hematology and Blood Transfusion, Prague, Czech Republic; 20 University of Heidelberg, Heidelberg, Germany; 21 Karolinska University Hospital, Stockholm, Sweden; 22 CHU de Lille, LIRIC, INSERM U995, Université de Lille, 59000 Lille, France

**Keywords:** Cancer epidemiology, Haematopoietic stem cells

## Abstract

The causes and rates of late patient-mortality following alloHCT for myelodysplastic syndromes or secondary acute myeloid leukemia were studied, to assess the contribution of relapse-related, treatment-related, and population factors. Data from EBMT on 6434 adults, who received a first alloHCT from January 2000 to December 2012, were retrospectively studied using combined land-marking, relative-survival methods and multi-state modeling techniques. Median age at alloHCT increased from 49 to 58 years, and the number of patients aged ≥65 years at alloHCT increased from 5 to 17%. Overall survival probability was 53% at 2 years and 35% at 10 years post-alloHCT. Survival probability at 5 years from the 2-year landmark was 88% for patients <45-year old and 63% for patients ≥65-year old at alloHCT. Cumulative incidence of nonrelapse mortality (NRM) for patients <45-year old at transplant was 7% rising to 25% for patients aged ≥65. For older patients, 31% of NRM-deaths could be attributed to population mortality. Favorable post-alloHCT long-term survival was seen; however, excess mortality-risk for all age groups was shown compared to the general population. A substantial part of total NRM for older patients was attributable to population mortality, information which aids the balanced explanation of post-HCT risk and helps improve long-term care.

## Introduction

The key motivation toward alloHCT for patients with myelodysplastic syndromes (MDS) is the expectation of long-term disease control or even cure. Data on long-term outcomes after alloHCT for MDS is still scarce however. Disease-specific long-term follow-up data have been published for patients with chronic myeloid leukemia, chronic lymphocytic leukemia, and in mixed cohorts dominated by patients with acute leukemias [1–5]. The absolute number and percentage of patients with MDS in these cohorts was relatively small; children were included and thus the median age of patients with MDS in these studies was below 35 years [3, 4, 6]. In consequence, these data might not reflect the contemporary population of adult patients with MDS who are referred for alloHCT [7–11].

In our study, we present a large cohort of adult patients with MDS who were registered with EBMT. We focus on two issues: first, we present 10-year outcomes after alloHCT from a very large group of patients, representing current practice in Europe. Second, we analyzed three types of mortality in one comprehensive model: general population mortality, relapse-related mortality, and treatment-related mortality (TRM). On the basis of this model we can show that for patients transplanted at an age ≥65 years, a significant proportion of total nonrelapse mortality (NRM) can be explained by general population mortality. For elderly patients we propose an estimate for TRM which is more accurate than assuming that TRM equals total NRM.

## Methods

The study population included all MDS patients who received a first alloHCT between January 2000 and December 2012, and who were recorded in the European Society for Blood and Marrow Transplantation (EBMT) registry. Patients were excluded if they fulfilled any of the following criteria: (a) had received cord blood as a stem cell source; (b) had a mismatched related or syngeneic donor; (c) were below the age of 18 years at alloHCT, or (d) where essential data were missing. Patients were also excluded if they originated from countries which contributed less than 25 patients to the dataset, or for which no population data were available in the Human Mortality Database. The dataset was closed in December 2016.

The endpoints of interest were overall survival (OS), event-free survival (EFS), relapse/progression, and NRM. OS was defined as the time from either alloHCT or landmark (LM) until death, with surviving patients censored at the moment of last follow-up. LM times were fixed at 2 and 5 years after alloHCT. At each LM, the analysis population consisted of either all patients still in follow-up (OS population) or all patients in follow-up without previous relapse/progression (EF population) (see Figure S1). EFS was defined as time to death or relapse/progression (whichever occurred first), with surviving patients censored at the last time-point they were reported disease-free. Its components, the cumulative incidences of relapse/progression (CIR) and NRM, were analyzed together by a competing risks model [12]. A multivariable Cox cause-specific hazards model was fitted to assess the dependence of the risk of NRM on year of alloHCT.

Probabilities of NRM and death after relapse of MDS were estimated on the basis of the data. We investigated which proportion of these probabilities could be considered as population mortality. Population mortality is defined as death due to causes acting in the general population from which the patient originates. This proportion could be estimated by assuming that the hazard (risk) for population mortality is the same for the patient population as for the general population. The hazard was calculated by the common method in relative survival, in which patients are matched by age, sex, and country in the year of alloHCT to a synthetic cohort from the general population, for whom survival information is available in the Human Mortality Database population tables (www.mortality.org) [13, 14]. Population NRM was subtracted from total NRM to obtain excess NRM. We used this as an approximation of TRM, defined as mortality which is related to either alloHCT (e.g., from graft-versus-host disease (GVHD)) or previous MDS treatment (e.g., resulting from iron overload). Due to the very poor prognosis associated with relapse/progression, the contribution of population mortality to death after relapse/progression was negligible. For this reason, death after relapse due to population reasons is not shown as a separate outcome while death after relapse and relapse-related mortality can be considered equivalent. All associated probabilities were estimated using a multistate model (see Supplementary material) [15].

We then fitted Cox proportional-hazards models for the excess hazard for death, defined as the difference between the observed hazard of the patient cohort and the hazard of the matched general population. Risk factors investigated were age, sex, year of alloHCT, MDS subclassification, conditioning, and donor. For the OS LM populations, previous relapse was added as a risk factor.

All analyses were performed in SPSS Version 23 and R 3.3.0 (https://cran.r-project.org/), packages “survival”, “cmprsk”, “prodlim”, “relsurv”, and “mstate”.

## Results

### Patient characteristics

Data of 6434 patients from 21 countries were analyzed. The number of transplants increased from 185 in 2000 to 862 in 2012. Median age at alloHCT increased from 49 years (range: 18–70 years) in 2000 to 58 years (18–76 years) in 2012 (Wilcoxon rank sum test, *p* < 0.001). During the same period, the number of patients who were 65 years or older at the time of alloHCT increased from nine (5% of all patients transplanted in that year) to 150 (17%). Totally, 21% of patients received alloHCT for MDS without excess blasts (EB), 42% for MDS with EB, and 37% for secondary acute myeloid leukemia (sAML) with a history of MDS. Patients with higher risk MDS subtypes were older at the time of alloHCT. For example, 29% of patients with MDS without EB were below 45-year old compared to 20% of patients with sAML, but only 8% of patients with MDS without EB were over 65-year old compared to 16% of patients with sAML (chi-square test, *p* < .001). The percentage of patients with unrelated donors increased from 35% in 2000 to 69% in 2012. Details on baseline patient characteristics are in Table 1.

**Table 1:** Patient characteristics at baseline

Parameter	Total number, *N* = 6434 [%]
*Patient sex*	
Male	59
Female	41
*Age at alloHCT*	
Median age (range) [y]	56 (18–76)
≤45 years [%]	21
45–55 years	25
55–65 years	41
>65 years	13
*Year of alloHCT*	
≤2002	11
2003–2004	10
2005–2006	14
2007–2008	18
2009–2010	21
2011–2012	27
*Patient Nationality*	
Belgium	4
France	13
Germany	31
Great Britain	13
Italy	9
The Netherlands	5
Spain	6
Remaining Countries*	19
*MDS subtype*	
MDS w/o excess blasts	21
MDS with excess blasts	42
Secondary AML	37
*Secondary origin (N * *= * *5016, 78%)*	
Preceding malignancy/autoimmune disease	19
*Interval diagnosis MDS-alloHCT*	
Median time (range) [y]	1 (0–43)
*Remission status at alloHCT*	
Complete remission	34
No complete remission	35
No remission attempt	31
*Karnofsky Status (N * *= * *4323, 67%)*	
90–100%	72
≤80%	28
*Previous auto HCT*	
No	99
Yes	1
*Donor match*	
HLA-identical sibling	41
Other donor	59
*Patient–donor sex constellation*	
Male-male	40
Male-female	19
Female-male	23
Female-female	17
*Conditioning*	
Myeloablative	44
Reduced Intensity	56
*Source of graft*	
Bone marrow	14
Peripheral Blood	86

*alloHCT* allogeneic hematopoietic stem cell transplantation, *y* year, *N* number, *MDS* myelodysplastic syndrome, *w/o* without, *AML* acute myeloid leukemia

Numbers and percentages  behind the variable name indicate number of patients with data for this variable, if less than 95% of data available; *countries contributing less than 3% patients to the study

### Completeness of follow-up

The median follow-up of survivors after alloHCT was 4.4 years. To assess the reporting quality during follow-up, we calculated the completeness of follow-up index (briefly, C-index), which gives the ratio of the total observed person-time of follow-up and the potential time of follow-up [16]. Overall, the reporting quality was good. The proportion of patients with complete data was 0.71. The median C-index for patients lost-to-follow-up was 0.56. The median C-index per center was 87% with an interquartile range from 53 to 99%. OS was not significantly different across centers with different reporting quality grouped by the C-index (log-rank test, *p* = 0.15).

As sensitivity analyses, the main analyses have been redone in the subgroup of patients transplanted in the period 2000-2008 (see Tables S1 and S2) to investigate if the inclusion of more recent patients biased the estimates of interest. For this earlier period, the median follow-up of patients alive at last follow-up was 7.6 years, the proportion of patients with complete data was 0.75 and the median C-index for patients with incomplete data was 0.58. Tables S1 and S2 show that the results were only marginally different from those performed on the whole cohort and would not lead to differences in interpretation. Therefore, results shown in this manuscript are based on the whole cohort.

We also investigated the impact of the risk factors included in our models, extended with center of transplantation, on censoring time and status, and found that only center, year of alloHCT and conditioning had a significant impact. For the last two variables, this is fully explained by the fact that patients transplanted more recently per definition have a shorter follow-up, and that reduced intensity conditioning was given more frequently in recent years. We investigated the impact of center on OS by means of a frailty model and found no significant impact. For these reasons, we conclude that the data allow to estimate reliable long-term outcomes.

### Outcomes from transplantation and of LM populations

#### Outcome from alloHCT

**Fig. 1:**
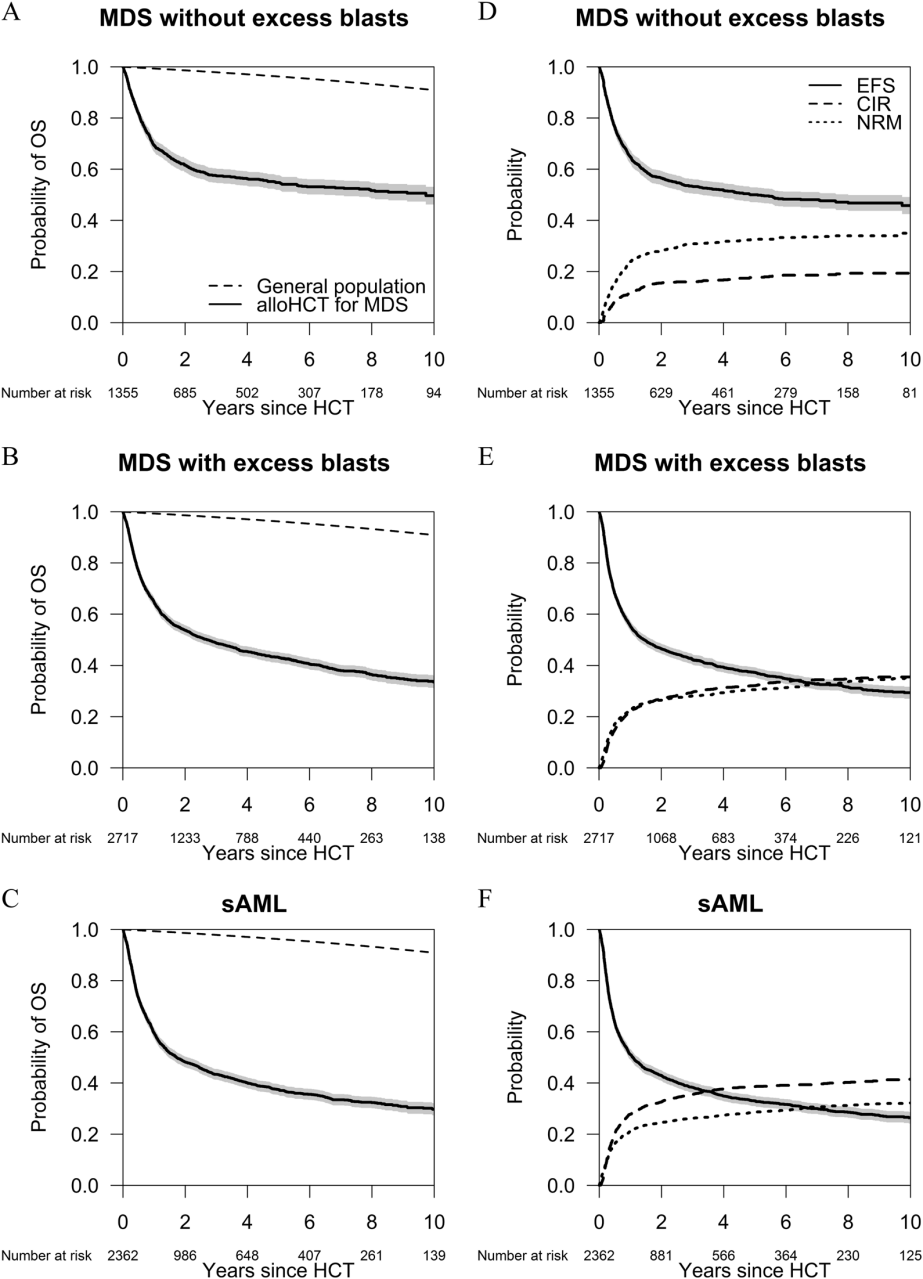
Long-term outcomes after alloHCT by subtype of MDS. **a**–**c** show the Kaplan–Meier plots for OS of three subtypes of MDS since alloHCT, with their 95% confidence intervals. The dashed lines show the survival probability of the general population matched by age, sex, country, and calendar year. **a** MDS without EB; **b** MDS with EB; **c** sAML. **d**–**f** show EFS of three subtypes of MDS since alloHCT with their 95% confidence intervals, and their components, the cumulative incidence of relapse/progression and of nonrelapse mortality. **d** MDS without EB; **e** MDS with EB; **f** sAML

For the whole cohort of patients, the probability of OS decreased from 53% (95% CI: 52–55%) at 2 years to 43% (95% CI: 42–45%) at 5 years and to 35% (95% CI: 34–37%) at 10 years after alloHCT. The probability of EFS at 2 years was 47% (95% CI: 46–49%) and decreased to 32% (95% CI: 30–33%) at 10 years. NRM and Relapse were causes of treatment-failure of the same magnitude. The NRM probability was 26% (95% CI: 25–27%) at 2 years and 34% (95% CI: 33–35%) at 10 years after alloHCT. Death after relapse was closely associated with the moment of relapse: the median survival after relapse was only 4.6 months (95% CI: 4.2–5.0 months). Two-year survival after relapse was 19% (95% CI: 17–21%). Plots for OS, EFS, CIR, and NRM since alloHCT by type of MDS are shown in Fig. 1.

The risk of NRM has decreased in recent years. In a multivariable model for the cause-specific hazard of NRM, a more recent year of transplantation was significantly associated with improved outcome (hazard ratio (HR) for 5 years difference 0.9, 95% CI: 0.8–1.0, *p* value = 0.002). Cumulative incidence of relapse was 27% (95% CI: 26–28%) at 2 years and 34% (95% CI: 33–36%) at 10 years after alloHCT.

The 5-year survival probability was lower with increasing age from 53% (95% CI: 50–56%) for patients <45 years of age at alloHCT, 46% (95% CI: 43–48%) for patients aged 45–55 years, 40% (95% CI: 38–42%) at ages 55–65 years, to 35% (95% CI: 32–39%) for patients above 65 years. Conversely, 5-year nonrelapse mortality was higher with increasing age: 25% (95% CI: 22–27%), 30% (95% CI: 28–32%), 32% (95% CI: 30–34%), and 36% (95% CI: 32–39%) for the respective age groups. Notably, the oldest patient alive at last follow-up (83 years) was 67-year old at transplant. The highest age reached in this cohort came from a patient who was 75 years old at transplantation and died aged 84 years. Survival estimates for patients with MDS with or without EB or secondary AML are provided in Table S3.

#### LM populations

Characteristics of patients still alive and in follow-up are in Table S4. Outcomes of LM populations were evaluated to focus on the long-term perspectives of patients who had survived the most risky, early period after alloHCT. Overall, the 1-year and 5-year risks of mortality for the 2-year OS LM population were 9% (95% CI: 8–10%) and 26% (95% CI: 24–28%). Separated by information on relapse between alloHCT and the 2-year LM, 2-year survivors who had experienced previous relapse had a 1-year mortality of 34% (95% CI: 29–39%), whereas 2-year event-free survivors had a 1-year mortality of 6% (95% CI: 5–7%). Information on outcomes of patients who passed the 2-year LM event-free is in Table 2.

**Table 2:** Outcomes of patients who passed the 2-year landmark event-free: overall and event-free survival, cumulative incidences of relapse/progression and non-relapse mortality by MDS subtype and age groups at 5 years since landmark

Classification		Overall survival	Event-free survival	Incidence of relapse/PD	Non-relapse mortality
		Point estimates at 5 years since landmark in % (95% CI)			
MDS subtype	MDS w/o EB	87 (84–90)	85 (82–88)	5 (3–8)	10 (7–12)
	MDS with EB	76 (73–80)	70 (67–74)	16 (13–19)	14 (11–17)
	sAML	74 (70–77)	70 (66–74)	16 (13–19)	14 (11–17)
Age group	<45 years	88 (86–91)	83 (80–87)	10 (7–13)	7 (4–9)
	45–55 years	79 (75–82)	75 (71–79)	14 (11–17)	12 (9–15)
	55–65 years	74 (71–78)	71 (67–74)	14 (12–17)	15 (12–18)
	≥65 years	63 (55–71)	56 (49–65)	19 (13–25)	25 (18–32)

*CI* confidence interval, *PD* progressive disease, *MDS* myelodysplastic syndrome, *EB* excess blasts, *w/o* without, *sAML* secondary acute myeloid leukemia

We evaluated the causes of death of all 676 patients whose death occurred more than 2-year post-alloHCT. Causes of death for patients who were deceased between 2 and 5 years after alloHCT are given in Table S5. Between 5 and 10 years, 208 deaths were reported, 104 of which were after relapse. Notably, even three patients (3%) of this subgroup died subsequent to the diagnosis of a second malignancy unrelated to MDS. In these three patients the secondary malignancy was adjudicated as primary cause of death by the local transplant physician.

NRM was also reported for 104 patients. Information on the main and contributing causes of death was available for 91 patients (87.5%). The main cause of death was directly related to alloHCT in 33 patients (36%). In these cases, GVHD was mentioned for 21 patients and infection for 14 patients as contributory cause of death. Secondary malignancies as the main cause of death were reported for 33 patients (36%). Among other main causes of death which were adjudicated to be independent from alloHCT for 21 patients, cardiovascular events (23%) were mentioned most frequently followed by neurologic diseases and chronic lung failure.

#### Treatment-related mortality and risk factors for excess mortality

**Fig. 2:**
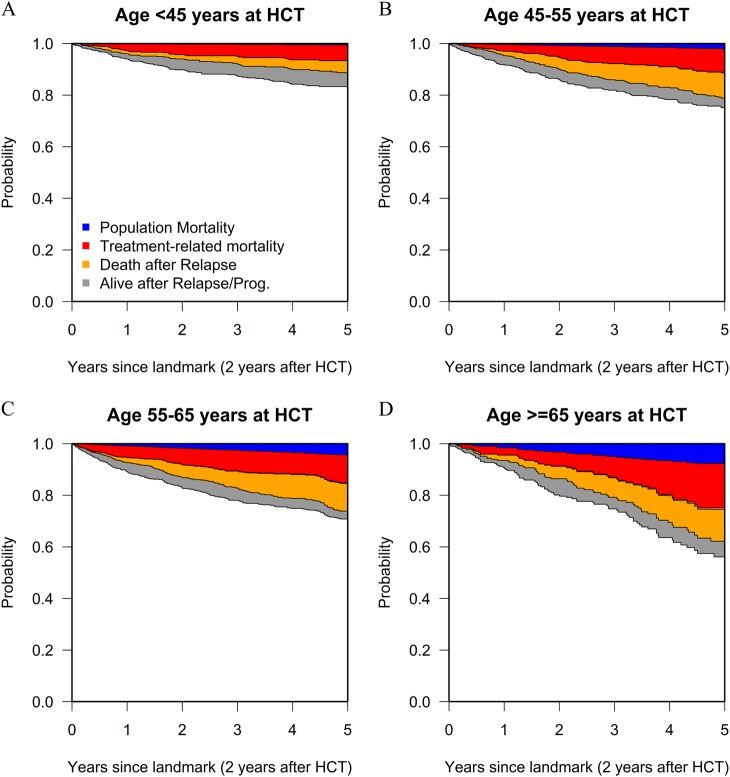
Mortality of the 2-year Landmark population by age groups. The plots show model-based probabilities of mortality by age groups due to different causes. These probabilities apply to patients still alive without relapse/progression at 2 years after alloHCT. The lower curve is the EFS curve; the curve above is the OS curve. The difference (gray area) between these two curves indicates the probability to be alive after relapse/progression. The observed non-relapse mortality has been split in two parts, based on mortality data for the general population: population mortality (blue area) and treatment-related mortality (red area). Death due to relapse is represented by the orange area. The model also incorporated the possibility of population mortality after relapse. Since its contribution is almost zero, it is not visible in the figures. Curves are stacked, meaning that the probabilities of the different outcomes are indicated by the distances between the lines

Overall, population mortality explained only a small part of early mortality after alloHCT. When we calculated different components of mortality for patients who were alive without previous relapse at the 2-year LM, we saw a different picture (see Fig. 2): for patients <45 years at alloHCT the estimated 5-year population mortality was 0.5% (95% CI: 0.4–0.6%) compared to 8% (95% CI: 7–8%) for patients who were ≥65 years at transplantation. The estimated 5-year TRM of these LM populations were 6% (95% CI: 4–8%) and 17% (95% CI: 10–25%) for these age groups, respectively. Combining this information shows that population mortality accounted for 8% of 5-year post-LM NRM for patients transplanted at an age <45 years, compared to 31% of NRM for patients transplanted at an age ≥65 years.

Finally, we performed multivariable analyses of risk factors for excess mortality. We confirmed the adverse impact of higher age on excess mortality (Table 3 and S6). For later LM populations (HR = 1.2, 95% CI: 1.1–1.3 and HR = 1.3, 95% CI: 1.1–1.6, for the 2- and 5-year LM OS population, respectively), this impact was higher than at HCT (HR = 1.1, 95% CI: 1.1–1.2). Female patients had a significantly better prognosis than male in the 5-year LM population (HR = 0.7, 95% CI: 0.5–1.0). Later calendar year of HCT was associated with better outcome in the first 2 years after HCT. We found a slightly worse outcome after the 2-year LM in more recent calendar years, possibly indicating that in recent years, less early mortality came at the expense of a somewhat increased later mortality, suggesting that for frail patients, cure had not been achieved. The more advanced stages of MDS kept their adverse prognostic impact over time, with even the highest HRs for the patients event-free at the 5-year LM (MDS with EB: 3.1, 95% CI: 1.4–7.0, *p* value = 0.005, sAML: 2.8, 95% CI: 1.2–6.3, *p* value = 0.01).

**Table 3:** Impact of risk factors in different time periods after alloHCT

Time-period/population	0–2 years, all patients		2–10 years, 2-year LM population		5–10 years, 5-year LM population	
Risk factor	HR (95% CI)	*p*-value	HR (95% CI)	*p-*value	HR (95% CI)	*p*-value
*Patient sex*						
Male	1		1		1	
Female	0.9 (0.9–1.0)	0.2	0.9 (0.7–1.0)	0.1	0.7 (0.5–1.0)	0.04
*Age at alloHCT (per decade)*	1.1 (1.1–1.2)	<0.001	1.2 (1.1–1.3)	<0.001	1.3 (1.1–1.6)	0.001
*Year of alloHCT (per 5 years)*	0.8 (0.8–0.9)	<0.001	1.2 (1.0–1.4)	0.02	1.4 (1.0–2.0)	0.06
*MDS subtype*						
MDS w/o EB	1		1		1	
MDS with EB	1.2 (1.1–1.4)	<0.001	1.9 (1.4–2.4)	<0.001	2.3 (1.3–4.0)	0.003
sAML	1.4 (1.2–1.5)	<0.001	2.1 (1.6–2.8)	<0.001	2.0 (1.2–3.6)	0.01
*Donor match*						
HLA-identical sibling	1		1		1	
Other donor	1.2 (1.1–1.3)	<0.001	1.1 (0.9–1.3)	0.3	1.1 (0.8–1.6)	0.5
*Conditioning*						
Myeloablative	1		1		1	
Reduced intensity	0.9 (0.9–1.0)	0.1	0.8 (0.7–1.0)	0.02	0.8 (0.5–1.1)	0.2
*Previous relapse*	NA		5.1 (4.2–6.1)	<0.001	5.0 (3.5–7.2)	<0.001

Cox models for excess mortality in defined time periods for patients alive at different landmarks.

*LM* landmark, *HR* hazard ratio, *CI* confidence interval, *NA* not applicable. Patients with missing information for conditioning were kept in the analysis in a separate category (not shown)

## Discussion

The 10-year event-free survival in this population of patients with MDS was 32% (95% CI: 30–33%). The observed rate of late relapse leveled off: in only 2% of patients with MDS and without EB and 3% of patients with sAML, relapse occurred between 5 and 10 years after alloHCT. These very low incidences of late relapses argue in favor of sustained immunologic disease control or complete eradication of the malignant founding clone after alloHCT.

Outcome after alloHCT improved during the 13-year period of treatment. We observed a risk reduction of 10% per 5-year period for excess mortality (HR = 0.9, *p* < 0.001). In line with data reported from one large center that compared past and recent outcomes during a 14-year period, this improvement was mainly due to a reduction of NRM [17].

Patients aged below 45 years at the time of alloHCT who passed the 2-year LM event-free have an excellent prognosis. Their excess mortality compared to the matched general population is relatively small (see Fig. 2a). The main strength of this study is, however, to show results of elderly patients, transplanted at a wide variety of centers. Altogether, 848 patients in this study were transplanted at an age above 65 years. Compared to existing publications on long-term survival, the median age of this cohort is more than 20 years higher [1, 3, 4]. The percentage of patients who were transplanted at an age ≥65 years increased from 4% in 2002 to 17% in 2012. Taking into account these rapid changes in patients referred for alloHCT, this cohort provides robust data on long-term survival of contemporary patients [9]. The importance of having such data is underscored by the notion that alloHCT is probably under-utilized in patients ≥65 years [10].

Current recommendations for alloHCT for patients with MDS state that fit patients without comorbidities should be considered for alloHCT regardless of their age [18]. Indeed, there is some controversy over the impact of age on transplant outcomes. McClune et al. [19] reported that age was no predictor for 2-year survival rates after alloHCT. However, it has been demonstrated more recently that even after adjustment for comorbidities and the performance status, age retains an impact on survival after alloHCT [20, 21]. We also found an impact of age on survival rates in our cohort of patients with MDS. However, in line with the publication from McClune et al. the impact of age on excess mortality was relatively small (HR = 1.1, 95% CI: 1.1–1.2) in the first 2 years after alloHCT. Notably, the impact of age on excess mortality increased for the 2-year (HR = 1.2, 95% CI: 1.1–1.3) and 5-year (HR = 1.3, 95% CI: 1.1–1.6) LM populations. The numbers given indicate average effects over all ages included in the study. For the oldest age group (>70 years at transplantation), not enough data were available to give a precise estimate of the incremental impact of higher age. Outside the setting of alloHCT, Della Porta et al. [22] integrated age into their age-adjusted WPSS and showed that higher age may lead to a shift to a higher risk category. Current data thus suggest that age should be taken into account when assessing the risk profile, but not used to preclude elderly patients from alloHCT [23–25].

NRM is usually regarded as an indicator of the risk of a transplant procedure. While the causality of alloHCT and transplant-specific complications such as GVHD can be established unequivocally, the definition of NRM by itself does not constitute a relationship between the treatment and the complication leading to death. At the individual level it is often impossible to assess whether a fatality was transplantation-related, pretreatment-related or independent. For example, GVHD may cause or aggravate endothelial damage and thus may increase the risk of cardiovascular disease, which is one of the most common causes of death in the general population. The same is true for secondary cancers diagnosed after alloHCT. The risk for certain cancers is increased after HCT, compared with the general population [26]. Yet, at the level of an individual patient, it is usually impossible to distinguish between a treatment-induced and a spontaneous solid cancer. The same holds true for cardiovascular disease and some infections for which treatment-induced and idiopathic events cannot be differentiated at patient level [27, 28]. To avoid subjective adjudication of causes of death, we chose a population-based approach. We assumed that patients with MDS had the same background risk of death from causes unrelated to the MDS and its treatment as the general population and that the excess risk of death was caused either directly or indirectly by the MDS and its treatment. Based on this assumption, we could split NRM into population mortality and excess NRM, where we interpreted the latter as TRM. We show that for patients transplanted at an age <45 years, total NRM is near-identical to TRM. However, for patients who are transplanted at an age ≥65 years, a substantial part of total NRM is population mortality, i.e., mortality which patients of that age inevitably face. Recent data suggest that patients with MDS are at greater risk of death from cardiovascular disease [29]. The percentage of cancer survivors may also be higher among patients with MDS than in the general population [30]. This implies that part of the excess NRM might not be TRM, but death due to morbidity associated with—but not caused by—MDS, and that true TRM might be somewhat smaller.

Huge efforts are being made to gain deeper insights into late complications after alloHCT and to improve long-term survivorship [31, 32]. Nevertheless, Rubinstein et al. [33] show that cancer survivorship services are not well integrated in primary care. A more precise approximation of treatment-related mortality by correcting for population mortality may help to define more realistic targets for cancer-survivor programs and to communicate more clearly with patients. It may already make a difference for a cancer-survivor to know that a substantial part of the risk of mortality is shared by an age- and sex-matched population of that country. In particular, for the growing number of elderly patients who are referred for alloHCT, the distinction between population mortality and true treatment-related mortality becomes increasingly important.

In conclusion, we propose that the consideration of population mortality should become standard, especially when long-term follow-up data after alloHCT are reported for elderly patients.

## Supplementary information

Supplementary information
