# Supplementary material for: Late treatment-related mortality versus competing causes of death after allogeneic transplantation for myelodysplastic syndromes and secondary acute myeloid leukemia
Source: Leukemia. 2018 Dec 20;33(3):686–95. doi: 10.1038/s41375-018-0302-y (PMC6756078; doi:10.1038/s41375-018-0302-y)
Supplement: Supplementary file 1 — Supplementary information [file 41375_2018_302_MOESM1_ESM.docx]

# Late Treatment-related Mortality versus Competing Causes of Death after Allogeneic Transplantation for Myelodysplastic Syndromes and Secondary Acute Myeloid Leukemia: Online Supplement

Running head: Long-term Survival after alloHCT for MDS

Johannes Schetelig^1,2,*^ Liesbeth C de Wreede^2,3,*^ Michel van Gelder^4^ Linda Koster^5^ Jürgen Finke^6^

Dietger Niederwieser^7^ Dietrich Beelen^8^ G.J. Mufti^9^ Uwe Platzbecker^1^ Arnold Ganser^10^ Silke Heidenreich^11^

Johan Maertens^12^ Gerard Socié^13^ Arne Brecht^14^ Matthias Stelljes^15^ Guido Kobbe^16^ Liisa Volin^17^

Arnon Nagler^18^ Antonin Vitek^19^ Thomas Luft^20^ Per Ljungman^21^ Ibrahim Yakoub-Agha^22^ Marie Robin^13^ Nicolaus Kröger^11^

*JS and LCdW contributed equally to this paper

^1^Universitaetsklinikum Dresden, Dresden, Germany.

^2^DKMS Clinical Trials Unit, Dresden, Germany

^3^Leiden University Medical Center, Leiden, The Netherlands.

^4^University Hospital Maastricht, Maastricht, The Netherlands.

^5^EBMT Data Office Leiden, Leiden, The Netherlands.

^6^University of Freiburg, Freiburg, Germany.

^7^University Hospital Leipzig, Leipzig, Germany.

^8^University Hospital, Essen, Germany.

^9^GKT School of Medicine, London, UK.

^10^Hannover Medical School, Hannover, Germany.

^11^University Hospital Eppendorf, Hamburg, Germany.

^12^University Hospital Gasthuisberg, Leuven, Belgium.

^13^Hopital St. Louis, Paris, France.

^14^Deutsche Klinik für Diagnostik, Wiesbaden, Germany.

^15^University of Münster, Münster, Germany.

^16^Heinrich Heine Universität, Düsseldorf, Germany.

^17^HUCH Comprehensive Cancer Center, Helsinki, Finland.

^18^Chaim Sheba Medical Center, Tel-Hashomer, Israel.

^19^Institute of Hematology and Blood Transfusion, Prague, Czech Republic.

^20^University of Heidelberg, Heidelberg, Germany.

^21^Karolinska University Hospital, Stockholm, Sweden.

^22^ CHU de Lille, LIRIC, INSERM U995, Université de Lille, 59000 Lille, France

**Corresponding author:**

Johannes Schetelig, MD, M.Sc.
Medizinische Klinik und Poliklinik I, Fetscherstraße 74, 01307 Dresden

T: +49-351-458-15604
F: +49-351-458-88-15604

Email: [Johannes.Schetelig@uniklinikum-dresden.de](mailto:Johannes.Schetelig@uniklinikum-dresden.de)

**Conflict of Interest:** The Authors declare no conflict of interest in this work.

# Supplement Online Only

## Figure S1. Analysis populations

Numbers of patients in follow-up at different time points after alloHCT. (1) Overall Survival (OS) population: all patients alive and in follow-up at the landmark; (2) Event-Free (EF) population: all patients in the OS population without relapse/progression before the landmark.


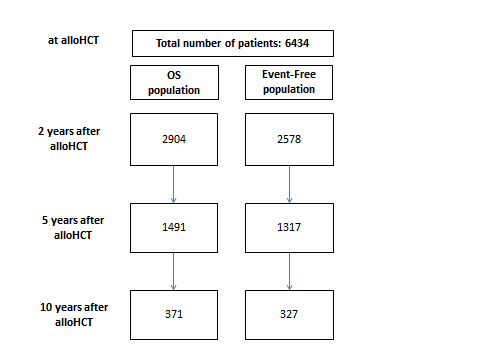


## Cox models for the excess hazard

The observed or total hazard of death can be split into two components: population hazard and excess hazard, or in formula: h_obs_(t) = h_pop_(t)+ h_exc_(t), where t is the time since alloHCT (0≤t<10 years). The population hazard is a function of sex, age, calendar year and country and is given by population tables. In formula: h_pop_(t)= h_pop_(t|sex, age, year, country). The excess hazard can be modelled by means of a Cox model, assuming proportional excess hazards for the different risk factors, comprising both population factors and factors specific to transplanted MDS patients. In formula: h_exc_(t)=h_0,exc_(t)exp(**β**’**Z**), where h_0,exc_(t) indicates the baseline excess hazard, **β** is the vector of regression coefficients and **Z**  is the vector of baseline covariates, in this case consisting of sex, age, year, MDS subtype, donor match, conditioning and previous relapse (the last only for the landmark models).

The regression coefficients and excess baseline hazard have been estimated by means of the Estève method which is based on a maximum likelihood approach. This has been implemented in the function ‘rsadd‘ in the package ‘relsurv’ in R (Pohar M, Stare J, Relative survival in R. Comput Methods Programs Biomed 81(3):272-278, 2006).

Investigation of the proportional hazards assumption of the excess hazard models has been performed by a test based on scaled partial residuals and further investigated by forming a Brownian bridge, as implemented in the functions ‘rs.zph’ and ‘rs.br’ in the relsurv package (Stare J, Pohar M, Henderson R, Goodness of fit of relative survival models, *Statistics in Medicine* 24:3911-3925, 2005). While we noted non-proportionality for several covariates in the model covering the whole period from 0 to 10 years, we split this interval into different parts to capture the time-dependent effect of these covariates (see Table 3).

## Methods for the estimation of treatment-related mortality

To estimate the contribution of population mortality to death without and after relapse/progression, a multi-state model was constructed (see Figure S2) (de Wreede LC, Fiocco M, Putter H: The mstate package for estimation and prediction in non- and semi-parametric multi-state and competing risks models. Comput Methods Programs Biomed 99:261-74, 2010). The model was a non-parametric Markov model stratified for age. It was an extension of the illness-death model with an intermediate state relapse/progression (State 2) and absorbing states population NRM (State 3), excess NRM (State 4), population death after relapse (State 5) and excess death after relapse (State 6). We assumed that the hazards for population mortality for the patient population were always the same as for the matched general population (Pohar Perme M, Esteve J, Rachet B: Analysing population-based cancer survival - settling the controversies. BMC Cancer 16:933, 2016; Pohar M, Stare J: Relative survival analysis in R. Comput Methods Programs Biomed 81(3):272-8, 2006) (in Figure S2: h_13_(t)=h_25_(t)). The hazard for excess NRM was then calculated by subtracting the population hazard from the observed NRM hazard at each event time-point. The hazard for excess death after relapse was calculated in the same way.

These hazards were then used as building blocks to calculate the transition probabilities in the multi-state model which yielded the quantities of interest, taking into account that for the patient population, the population hazards did not translate into the same death probabilities as for the general population due to the competing risks of disease- and treatment related failures. The sum of population NRM and excess NRM was equal to (observed) total NRM, and the same held for death after relapse. Standard errors were calculated by means of bootstrapping.

In our study, we interpreted excess NRM as a very good estimate for Treatment-Related Mortality (including direct and indirect consequences of pre-treatment and alloHCT). Early mortality after relapse was high across all age groups. As a consequence, population mortality after relapse was negligible for all age groups and excess death after relapse almost equaled total relapse-related mortality. Although we accounted for population mortality after relapse in the model and plots, we did not report the probabilities in the text. We reported population NRM as population mortality and interpreted excess death after relapse as a very good estimate for relapse-related mortality.

This part of the analysis was performed in R 3.3.0 (https://cran.r-project.org/), packages ‘survival’, ‘relsurv’, and ‘mstate’.

## Figure S2. A multi-state model incorporating population mortality

All patients start in the state alive event-free after alloHCT. They can progress to relapse/progression and death. The term “*h”* indicates the hazard of making a transition from one state to the next, equivalent to experiencing an event. The probability to remain in State 1 at time *t* is equivalent to EFS(*t*). The sum of the probabilities to be in any of the death states (States 3-6) at time *t* adds up to 1-OS(*t*).

## Table S1. Outcomes of patients who passed the 2-year Landmark event-free: Overall and Event-Free Survival, Cumulative Incidences of Relapse/Progression and Non-Relapse Mortality by MDS subtype and age groups at 5 years since Landmark

| **Classification** |  | **Overall Survival** | **Event-Free Survival** | **Incidence of Relapse/PD** | **Non-Relapse Mortality** |
| --- | --- | --- | --- | --- | --- |
|  |  | Point estimates at 5 years since landmark in % (95%-CI) | | | |
|  | MDS w/o EB | 88 (85-92) | 87 (83-90) | 5 (3-8) | 8 (5-11) |
| MDS Subtype | MDS with EB | 79 (75-83) | 73 (69-77) | 15 (11-18) | 13 (10-16) |
|  | sAML | 75 (72-79) | 73 (69-77) | 14 (11-17) | 14 (11-17) |
| Age Group | <45 years | 87 (84-91) | 83 (79-86) | 10 (7-13) | 8 (5-10) |
|  | 45 – 55 years | 81 (77-85) | 77 (73-81) | 12 (9-16) | 11 (8-14) |
|  | 55 – 65 years | 76 (72-80) | 74 (70-78) | 13 (10-16) | 14 (11-17) |
|  | ≥ 65 years | 64 (55-75) | 57 (48-69) | 17 (9-25) | 26 (17-35) |

*Abbreviations: CI, confidence interval; MDS, myelodysplastic syndrome; EB, excess blasts; w/o, without; sAML, secondary Acute Myeloid Leukemia.*

The table shows the analysis reported in Table 2 redone in the restricted cohort of patients transplanted in the period 2000-2008. Outcomes are slightly better than for the whole cohort which is in line with our findings that for the 2-year landmark population, outcomes have marginally deteriorated over the years – in contrast to the first two years after alloHCT where outcomes have improved.

## Table S2. Impact of risk factors in different time periods after alloHCT

##

| **Time-Period / Population** | **0-2 years,  All Patients** | | **2-10 years,  2-year LM population** | | **5-10 years,  5-year LM population** | |
| --- | --- | --- | --- | --- | --- | --- |
| **Risk factor** | HR (95%-CI) | p-value | HR (95%-CI) | p-value | HR (95%-CI) | p-value |
| Patient sex  Male  Female | 1  1.0 (0.9-1.1) | 0.7 | 1  0.8 (0.6-1.0) | 0.04 | 1  0.6 (0.4-1.0) | 0.03 |
| Age at alloHCT (per decade) | 1.1 (1.1-1.2) | <0.001 | 1.2 (1.1-1.4) | <0.001 | 1.3 (1.0-1.6) | 0.02 |
| Year of alloHCT (per 5 years) | 0.8 (0.7-0.9) | <0.001 | 1.1 (0.9-1.4) | 0.4 | 1.3 (0.8-2.1) | 0.3 |
| MDS subtype  MDS w/o EB  MDS with EB  sAML | 1  1.2 (1.1-1.4)  1.3 (1.1-1.5) | <0.001  <0.001 | 1  1.9 (1.3-2.7)  2.1 (1.5-3.0) | <0.001  <0.001 | 1  2.5 (1.3-4.9)  2.0 (1.0-3.9) | 0.006  0.05 |
| Donor Match  HLA-identical Sibling  Other donor | 1  1.2 (1.1-1.4) | <0.001 | 1  1.2 (1.0-1.5) | 0.08 | 1  1.3 (0.9-1.9) | 0.2 |
| Conditioning  Myeloablative  Reduced Intensity | 1  1.0 (0.9-1.1) | 1.0 | 1  0.8 (0.6-1.0) | 0.03 | 1  0.8 (0.5-1.3) | 0.4 |
| Previous relapse | NA |  | 4.8 (3.8-6.2) | <0.001 | 5.1 (3.4-7.7) | <0.001 |

*Abbreviations: LM, landmark; HR, hazard ratio; CI, confidence interval; alloHCT, allogeneic hematopoietic stem cell transplantation; MDS, myelodysplastic syndrome; EB, excess blasts; w/o, without; sAML, secondary Acute Myeloid Leukemia; HLA, human leukocyte antigen; NA, not applicable.*

Cox models for excess mortality in defined time periods for patients alive at different landmarks. The table shows the analysis reported in Table 3 redone in the restricted cohort of patients transplanted in the period 2000-2008.

## Table S3. Overall and Event-Free Survival, Cumulative Incidences of Relapse/Progression and Non-Relapse Mortality for patients with MDS without Excess Blasts (EB), with EB and with secondary AML at 2, 5 and 10 years after allo-HCT

| **Time from alloHCT** | **MDS Subtype** | **Overall Survival** | **Event-Free Survival** | **Incidence of Relapse/PD** | **Non-Relapse Mortality** |
| --- | --- | --- | --- | --- | --- |
|  |  | Percent (95%-CI) | | | |
| 2 years | MDS w/o EB | 62 (59-64) | 57 (54-59) | 15 (13-17) | 28 (26-31) |
|  | MDS with EB | 54 (52-56) | 47 (45-49) | 27 (25-29) | 26 (25-28) |
|  | sAML | 48 (46-50) | 43 (41-45) | 33 (31-35) | 25 (23-26) |
| 5 years | MDS w/o EB | 55 (52-58) | 50 (47-53) | 18 (15-20) | 32 (30-35) |
|  | MDS with EB | 43 (41-45) | 37 (35-39) | 32 (30-34) | 31 (29-32) |
|  | sAML | 37 (35-40) | 33 (31-35) | 39 (37-41) | 28 (27-30) |
| 10 years | MDS w/o EB | 50 (46-53) | 46 (43-49) | 19 (17-22) | 35 (32-38) |
|  | MDS with EB | 34 (31-36) | 29 (27-32) | 36 (33-38) | 35 (33-37) |
|  | sAML | 30 (27-32) | 26 (24-29) | 41 (39-44) | 32 (30-34) |

*Abbreviations: alloHCT, allogeneic hematopoietic stem cell transplantation; CI, confidence interval; PD, progressive disease; MDS, myelodysplastic syndrome; EB, excess blasts; w/o, without; sAML, secondary Acute Myeloid Leukemia.*

## Table S4. Patient Characteristics at Baseline and for 2-year and 5-year survivors

| **Parameter** | **Total** | **All patients**  **[%]**  **N=6434** | **2y OS LM population**  **[%]**  **N=2904** | **5y OS LM population**  **[%]**  **N=1491** |
| --- | --- | --- | --- | --- |
| **Patient Sex** | Male | 59 | 57 | 57 |
|  | Female | 41 | 43 | 43 |
| **Age at alloHCT** | median (range) [years] | 56 (18-76) | 54 (18-76) | 53 (18-75) |
|  | ≤45years [%] | 21 | 24 | 28 |
|  | 45-55 years | 25 | 26 | 28 |
|  | 55-65 years | 41 | 38 | 35 |
|  | >65years | 13 | 12 | 9 |
| **Year of alloHCT** | ≤2002 | 11 | 11 | 17 |
|  | 2003 – 2004 | 10 | 10 | 16 |
|  | 2005 – 2006 | 14 | 15 | 21 |
|  | 2007 – 2008 | 18 | 18 | 24 |
|  | 2009 – 2010 | 21 | 20 | 19 |
|  | 2011 – 2012 | 27 | 25 | 3 |
| **Patient Nationality** | Belgium | 4 | 3 | 3 |
|  | France | 13 | 14 | 11 |
|  | Germany | 31 | 31 | 31 |
|  | Great Britain | 13 | 13 | 13 |
|  | Italy | 9 | 11 | 14 |
|  | The Netherlands | 5 | 5 | 6 |
|  | Spain | 6 | 4 | 4 |
|  | Remaining Countries* | 19 | 18 | 18 |
| **MDS subtype** | MDS w/o excess blasts | 21 | 24 | 26 |
|  | MDS with excess blasts | 42 | 42 | 40 |
|  | Secondary AML | 37 | 34 | 34 |
| **Secondary Origin (N=5016, 78%)** | Preceding malignancy / autoimmune disease | 19 | 17 | 17 |
| **Interval diagnosis  MDS-alloHCT** | median (range) [years] | 1 (0-43) | 1 (0-43) | 1 (0-43) |
| **Remission status at alloHCT** | Complete Remission | 34 | 35 | 33 |
|  | No Remission attempt | 31 | 35 | 41 |
|  | No Complete Remission | 35 | 31 | 27 |
| **Karnofsky Status  (N=4323, 67%)** | 90-100% | 72 | 77 | 78 |
|  | ≤80% | 28 | 23 | 22 |
| **Previous autoHCT** | No | 99 | 99 | 99 |
|  | Yes | 1 | 1 | 1 |
| **Donor Match** | HLA-identical Sibling | 41 | 44 | 49 |
|  | Other donor | 59 | 56 | 51 |
| **Patient-Donor Sex constellation** | Male-male | 40 | 39 | 40 |
|  | Male-female | 19 | 18 | 18 |
|  | Female-male | 23 | 25 | 24 |
|  | Female-female | 17 | 18 | 19 |
| **Conditioning** | Myeloablative | 44 | 44 | 48 |
|  | Reduced Intensity | 56 | 56 | 52 |
| **Source of graft** | Bone marrow | 14 | 14 | 16 |
|  | Peripheral Blood | 86 | 86 | 84 |
| **T-cell depletion (TCD) (N=5569, 87%)** | ATG/Alemtuzumab | 58 | 58 | 53 |
|  | Ex vivo TCD | 5 | 4 | 6 |
|  | No TCD | 37 | 38 | 41 |
| **Patients with Relapse before Landmark** |  | - | 11 | 12 |
| **Follow-up since alloHCT for patients alive at last follow-up** | median (range) [years] | 4 (0-17) | 6 (2-17) | 8 (5-17) |

*Abbreviations: OS, overall survival; LM, landmark; N, number; alloHCT, allogeneic hematopoietic stem cell transplantation; MDS, myelodysplastic syndrome; w/o, without; AML, acute myeloid leukemia; autoHCT, autologous hematopoietic stem cell transplantation; HLA, human leukocyte antigen; ATG, anti-thymocyte globulin.*

Legend: Numbers and percentages provided in the first column indicate number of patients with data for this variable at baseline, if less than 95% of data available; *countries contributing less than 3% patients to the study. The patient characteristics at baseline, at the 2-year LM and the 5-year LM were different. These differences partly reflect the changes of patient selection over time as outlined in the first paragraph. However, they also reflect a selection process during follow-up after alloHCT, implying that high-risk patients died earlier and did not reach the respective LMs, which is for instance indicated by the decreasing median age and the increasing proportion of patients with MDS without EB over time.

## Table S5. Causes of Death between 2 and 5 years after Transplantation

| Classification of Main Cause and contributing Causes of Death | Numbers (%) |
| --- | --- |
| Death after Relapse | 276 (59) |
| Non-Relapse Death | 192 (41) |
| Subclassification of contributing Causes of Non-Relapse Death* | 192 (100) |
| Graft versus Host Disease | 62 (32) |
| Infections | 59 (31) |
| Secondary Malignancy | 27 (14) |
| Interstitial Pneumonitis / Pulmonary Toxicity | 12 (6) |
| Cardiac events | 10 (5) |
| Neurologic events | 9 (5) |
| Hemorrhage | 5 (3) |
| Kidney failure | 5 (3) |
| Multiorgan failure | 15 (8) |
| Unknown | 24 (13) |

Legend: * more than one contributing cause of death could be reported for a patient, percentages thus add up to more than 100%

## Table S6. Cox model for excess mortality

| **Time-period/population** | **0-10 years/All patients** | |
| --- | --- | --- |
| **Risk factor** | **HR (95%-CI)** | **p-value** |
| Patient sex  Male Female | 1 0.9 (0.9-1.0) | 0.09 |
| Age at alloHCT (per decade) | 1.1 (1.1-1.2) | <0.001 |
| Year of alloHCT (per 5 years) | 0.9 (0.8-0.9) | <0.001 |
| MDS subtype  MDS w/o EB MDS with EB sAML | 1 1.3 (1.2-1.5) 1.5 (1.3-1.6) | <0.001 <0.001 |
| Donor Match  HLA-identical Sibling Other donor | 1 1.2 (1.1-1.3) | <0.001 |
| Conditioning  Myeloablative Reduced Intensity | 1 0.9 (0.9-1.0) | 0.08 |

*Abbreviations: HR, Hazard Ratio; CI, Confidence Interval; alloHCT, allogeneic hematopoietic stem cell transplantation; MDS, myelodysplastic syndrome; EB, excess blasts; w/o, without; sAML, secondary Acute Myeloid Leukemia; HLA, human leukocyte antigen.*

Patients with missing information for conditioning were kept in the analysis in a separate category (not shown).
